# “Choose Your Own Adventure”: Web-Based Case Studies of Inclusive Education as a Form of Professional Learning for School Principals

**DOI:** 10.1177/19427751211046978

**Published:** 2021-10-01

**Authors:** Steve Sider, Kimberly Maich, Jacqueline Specht, Carolyn Treadgold, Hillary Winger

**Affiliations:** 1 Wilfrid Laurier University, Waterloo, ON, Canada; 2 Memorial University of Newfoundland, St. John’s, Canada; 3 University of Western Ontario Faculty of Education, London, Canada; 4 Ontario Principals’ Council, Whitby, Canada; 5 Wilfrid Laurier University Faculty of Education, Waterloo, ON, Canada

**Keywords:** case study approach, professional development, principal preparation, special education, leadership and learning

## Abstract

We examine the process of developing web-based case studies, a novel form of professional learning for principals, specifically related to inclusive school leadership. Based on the input from 39 principals, 5 case studies were developed with branching scenarios that provided multiple options for decision-making. These “choose your own adventure” case studies were used in a special education for school administrators course with 109 participants in Ontario, Canada. We consider the authenticity of the cases, the importance of incorporating multiple perspectives, and issues related to function, form, and choice. We incorporate five lessons for developing web-based case studies.

This study provides an opportunity to address gaps in the academic literature by examining how web-based case studies on inclusive school leadership, developed for school principals,^1^ can be used for their professional learning in supporting students with special education needs. The study explores the following research question: *How can web-based case studies provide an effective form of learning for school principals in a professional learning course on special education?* We developed and evaluated five online case studies that were used in a professional development course for principals in Ontario, Canada. The 125-hour course *Special Education for Administrators Qualification Program* was offered by the Ontario Principals’ Council (OPC) through a fully online mode of delivery. The OPC is a professional association representing more than 5,000 principals and vice-principals in Ontario’s publicly-funded school system and is responsible for principal qualifications and professional learning. The course was completed by 109 principals and vice-principals from across Ontario. The cases were provided via an online platform with branching scenarios that included multi-modal delivery mechanisms (i.e., text, video, and images), providing participants with multiple options for decision-making. These branching scenarios offered a “choose your own adventure” form of professional learning.

This article presents a description of the process of development and delivery of these five case studies and explores this form of an experiential learning model for principals’ professional learning. This study builds on, and extends, the work of Tucker and Dexter (2011) who call for further examination of online cases in the professional development of school administrators. We utilized an iterative research design in which input from school leaders informed the development of the case studies which were then piloted with 109 principals in a professional learning course. In following this design process, we worked with school administrators in the field who could identify the problems, solutions, and refinements based on their experience (Cosner, 2019). Within this framework, we designed the cases, implemented them, and utilized feedback from participants to adjust the cases, as needed.

## Literature Review

### School Principals and Students With Special Education Needs

Over the past 30 years, school jurisdictions in Canada have supported teachers in adaptations to meet the needs of a wide variety of students (McCrimmon, 2015; Specht et al., 2016). Limited scholarly attention, however, has been given to the role of school principals in supporting inclusive schools (Cobb, 2015; Sider et al., 2017). Although inclusive education can refer to diversity broadly, in this article we focus specifically on students with special education needs (SEN). In inclusive schools, students with SEN have equitable access to education and are given supports for successful learning in their neighborhood schools in age-appropriate classrooms (Specht et al., 2016). Principals are also special education leaders and determine approaches—and attitudes—around the inclusion of students with SEN (DeMatthews et al., 2020, 2021). As school leaders, principals have central roles in developing healthy school environments that foster success of all students—and educators (Leithwood et al., 2010). School leaders build and foster relationships with students, parents, teachers, and other members of the school community (Cobb, 2015). Along with teachers, they play a large role in the academic success of students (Davis & Darling-Hammond, 2012). Approximately 15% of students have been identified with SEN in Canada; in Ontario alone, Canada’s largest province with a total population of approximately 14.5 million people, more than 240,000 students require some form of special education support in regular classrooms (Bennett et al., 2019). As a result, supporting students with SEN in inclusive schools is a significant aspect of any principal’s role.

Sider et al. (2017) outline key day-to-day responsibilities of principals supporting students with SEN. These responsibilities include communicating with teachers, parents and caregivers, and support staff about specialized areas of need, leading meetings to implement and/or review supports, interacting with students and their family members, and providing professional learning support to the full school team about inclusive education. Cobb (2015) also provides insight into the roles of school principals in supporting students with SEN by exploring aspects such as advocate, organizer, visionary, partner, coach, and conflict resolver.

New principals often report stress and trauma during their first years in their leadership role (Slater et al., 2018) and discuss a feeling of under-preparedness for the profession (Campanotta et al., 2018). Matthews and Crow (2010) state that the principalship literature “has emphasized an instructional leadership role, in which principals are expected to focus on teaching and learning and facilitate the learning community of the school. However, few writers have emphasized the principal as a learner” (p. 65). Recent research has investigated the necessary components in effective courses that focus on the professional learning of new principals (Campanotta et al., 2018; Davis & Darling-Hammond, 2012; Slater et al., 2018). Among the recommendations are: having a strong connection between theory and practice; using active learning; focusing on instruction and school improvement; and, accessing people who have been administrators working in schools (Johnson et al., 2020; Wang et al., 2018).

School principals are tasked with supporting students with SEN in their schools. There are, however, limited professional learning resources available to help school leaders know *how* to effectively include all students (Sider et al., 2017). As a result, “leadership program faculty must begin to prepare leaders to engage underserved and marginalized communities and build strong structures able to support the needs of diverse populations of students” (Johnson et al., 2020, p. 27). Case studies are a form of professional learning which can provide authentic narratives and associated resources to help principals with decision-making and problem-solving.

### Case Studies for Professional Learning

Case studies provide a mechanism to effectively engage educators—including school principals—in professional development (Yin, 2013). They have a “rich, strong history as an effective, engaging student-centered instructional strategy to supplement more traditional approaches to knowledge transmission” (Maich, 2015, p. xiv) that help stories of humanity to come alive in a context of insightful, reflective meaning-making (Bano et al., 2015; Kantar & Massouh, 2015). Popil (2011) suggests that case studies can promote critical thinking by providing opportunities to “apply theory to practice, practice decision making skills, use different viewpoints, engage in data analysis, and synthesize course content” (p. 205). They also provide opportunities to compare, connect, critique, judge, defend, analyze, and prioritize (Davis & Wilcox, 2003; Foran, 2001). Sherman (2008) specifically promoted the use of cases to allow principals to visualize the realities of the role and successful resolution to situations that arise.

Cases are used in teacher preparation and professional development (Judge et al., 2013; Vereb et al., 2015) as well as, more specifically, providing insight into supporting students with SEN (Maich & Hill, 2018). Engaging in case-based learning, and reflecting upon such cases, can make significant contributions to the professional learning experience of educators (Howie & Bagnall, 2013)—including school leaders—when and where direct observation is not desirable nor possible (Maich & Hill, 2018, p. x). Case studies may be particularly effective tools in principals’ training because they often involve ethical decision making, identifying options with limited information, and considering contextual nuances including the pressures that principals encounter (DeMatthews & Serafini, 2019). Web-based case studies with branching scenarios may provide active learning opportunities which integrates theory and practice in helping principals develop their leadership competencies in meeting the needs of all students (DeMatthews et al., 2020; Dexter et al., 2020).

This research study introduces a novel form of online case study previously used in medical education (Beck et al., 2017; Burke, 2017) but appears largely absent from the related scholarly literature in school-based practice, including educational leadership. Aside from research by Tucker and Dexter (2011) and Dexter et al. (2020), there is limited research examining the use of web-based case studies in education. Dexter et al. (2020) outline how interactive learning tools, such as digital cases, can support school leadership development through immersive learning. Similarly, this study explores the development of case studies that incorporate web-based branching options and scenarios for active-choice making that simulates professional decision-making.

Web-based case studies which include branching options are sometimes referred to as “choose your own adventure” cases (Beck et al., 2017). Such case studies provide text-based accounts of issues or situations, including related contextual information. In addition, they also provide branching scenarios, providing users with opportunities to select preferred strategies, options, information, and resources to assist decision-making. These branching scenarios offer a more authentic, closer-to-reality experience than traditional text-based case studies which tend to be linear and sequential without consideration for multiple options (Dexter et al., 2020).

An important theoretical aspect of this study which informs the use of immersive case study is Kolb’s (1984) experiential learning theory where learning is a “process whereby knowledge is created through the transformation of experience” (p. 38). The cyclical nature of Kolb’s experiential learning theory is typically conceptualized as an interactive process in which reflecting upon concrete experiences leads to new conclusions and ideas. This new learning then leads to new ideations for further experiences. Kolb viewed this form of learning as an integration of concrete emotional experiences with cognitive processes (Kolb & Fry, 1975). Kolb argued that “learning is best facilitated in an environment where there is a dialectic tension and conflict between immediate, concrete experience and analytic detachment” (Kolb & Fry, 1975, p. 35). The branching scenarios of the cases developed in this study provides an opportunity for principals to analyze multiple options for decision-making.

Case studies have been examined as a form of experiential learning (e.g., Arseven, 2018) but the novelty of web-based case studies with branching options presents an important new area of study for experiential learning theory. Web-based case studies provide an opportunity to use a case as a representation of real-life situations as the concrete experience central to experiential learning theory. Kolb argued that, “in the process of learning one moves in varying degrees from actor to observer, and from specific involvement to general analytic detachment” (Kolb & Fry, 1975, p. 36). Web-based case studies enable participants to make connections with their own experiences while also fostering opportunities to analyze the case from the perspective of an observer. Being able to reflect on cases, make connections to personal contexts, and inform practices going forward aligns with experiential learning theory. Engaging in case studies that provide multiple options and with technology-enhanced aspects facilitated by an online platform provides such an opportunity to add an experiential aspect to traditional case studies.

## Method

In this study, we used an emergent design research framework in which the development and validation of the cases is part of the descriptive analysis of their use and perceived effectiveness (Plano Clarke & Creswell, 2015). The research project was divided into three phases during which all research ethics protocols were adhered to in keeping with university clearances. Participants in all phases of the study were not asked to provide information such as gender, whether they identified as being racialized, or years of experience. In the first phase of the study, the research team collected questionnaire and interview data from 39 school principals in Ontario, Canada to better understand the issues that principals identified as important in their professional responsibilities around supporting students with SEN. These responses were examined, coded, and categorized using principles of thematic analysis (Braun & Clarke, 2006). This process involved manual coding of questionnaire and interview transcript data sets. Relevant codes were grouped into themes, as defined by Braun and Clarke (2006) as that which “captures something important about the data in relation to the research question, and represents some level of patterned responses or meaning within the data set” (p. 82). The themes then helped frame the focus areas of the five case studies that were written as the second phase of the study. Narratives from these case studies were based on the responses of the principals in the first phase of the study. Supplementary resources, including videos of principals responding to these case studies, were added to the case narratives on a dedicated website. In the third phase of the study, 109 participants in a professional learning course on special education and school leadership used the case studies as part of their instructional materials. Participants provided feedback on the effectiveness of the case studies as part of an interactive process of improving the cases.

### Phase 1: Data Collection and Analysis

The research team designed and distributed a questionnaire to examine how instructors of an OPC Principal Qualifications Program course perceived the inclusion of students with SEN in schools. The participants were asked to identify key issues in their work to support students with SEN in inclusive schools. The questionnaire was completed online and data stored on a secure server. Thirty-nine instructors responded to the questionnaire. Respondents were current and former principals in Ontario elementary and secondary school settings (see Table 1 for demographic information). Respondents were asked if they would consider participating in a follow-up interview to provide further insights into special education and inclusive school leadership for the development of the case studies. As a result, six telephone interviews of approximately 20 minutes each were conducted. All interviews were digitally recorded and transcribed. Interviewees were sent a copy of the transcript for member checking to ensure accuracy (Mero-Jaffe, 2011).

**Table 1.** Demographic Information on Principal Qualifications Program (PQP) Instructors.

Number of instructors working in an elementary or secondary school setting	Elementary school: 20
	Secondary school: 6
	Not currently working in a school setting: 13
Number of years instructors have been employed by a school board/district	11–15 years: 2
	16–20 years: 9
	20+ years: 28
Number of years instructors have been school principals	1–5 years: 3
	6–10 years: 15
	11–15 years: 17
	16–20 years: 3
	20+ years: 1

An emergent approach that identified frequently used words from the questionnaire and interview responses was used in the data analysis in the first phase of the study. From these key words, categories of experiences that principals had with students with SEN were established by the research team. The research team interviewed two staff members from the OPC responsible for special education and principals’ professional learning as part of a validation activity to ensure the identified categories of experiences aligned with the focus areas these experts had identified through their experiences in special education and school leadership (Clayton, 1997). From this exercise, five major focus areas were identified: teacher efficacy, resource realities, parent engagement, mental health of school administrators, and problematic student behaviors.

### Phase 2: Case Study Development

Phase 2 study involved the writing, revising, and validating exercise phase of the study. Five case studies on inclusive school leadership were drafted, each reflecting one of the identified focus areas. The narratives for the case studies, as well as the branching scenarios, were based on the data collected through the questionnaires and interviews from the first phase of the study. Each case followed a similar format with narrative text which provided the background and emerging issues of the case. As well, each case hinged on a critical experience focused on the case’s hypothetical principal. Four branching options were provided in each case. Branching options were not meant to provide preferred and incorrect options but rather four potential outcomes of each case. The cases were typically 800 to 1,000 words in length. Supplementary resources were identified to accompany each case.

The research team completed an internal validation exercise to ensure the cases accurately reflected the identified focus areas and experiences from the first phase of the study. This validation included having each case reviewed by four members of the research team and a member of the special education leadership team at the OPC. After the review and subsequent editing, an external validation exercise was completed. Each of the revised cases were reviewed by two current principals to ensure the cases authentically reflected their experiences as principals. Based on this input, further revisions were completed and the final five case studies were completed (see Table 2 for case summaries).

**Table 2.** Case Titles, Focus Areas, and Descriptions.

Case title	Case focus area	Case description
Case 1: Karter’s Writing: A Case of Teacher Efficacy	Teacher efficacy	A new teacher seeks support at a staff meeting for a student. The principal must decide how to support the teacher and the student.
Case 2: Ms. Lee’s Decision: A Case of Funding Changes for Students with Autism	Resource realities	A principal delivers news to staff about changes to specialized therapy funding. The principal must decide how to respond to the concerns of staff.
Case 3: A Co-Op Placement for Charlotte	Parent engagement	A high school vice-principal must decide how to respond to a parent’s request for his daughter with Down Syndrome to participate in a co-op placement.
Case 4: “I’m Struggling. . .”	Mental health of school administrators	A teacher approaches a school principal to discuss the ways he is struggling to keep up with the demands of his job particularly related to supporting students with special education needs. The principal must decide how to support the teacher’s mental well-being.
Case 5: Ms. Morvan’s Decision: A Case of Student Oppositional Behaviour and Inclusive School Culture	Problematic school behaviors	An elementary school principal must decide how to respond when a student with Autism Spectrum Disorder becomes aggressive with another student.

The research team completed video vignettes with ten experienced school principals who agreed to provide commentaries related to the cases. In advance of video recording, principals were sent a case to review and questions to consider. Principals were filmed for approximately 30 minutes as they articulated their responses to the case. These video files were reviewed by the research team and were edited into two or three vignettes of approximately 2 minutes each that could be used as supplementary resources to the cases.

The research team investigated different online platforms to host the case studies and decided upon Articulate Rise, an online management system. A research assistant worked with a digital media specialist to develop the cases into an online format and to ensure online visual and text consistency among the cases. Once the cases were posted online, access was provided to the full research team to review the cases. Feedback from this final review stage was provided to the digital media specialist and a finalized online version of the cases was made available. See Appendix for screen shot of the cases including the resources. The cases are available open access at https://www.leadtoinclude.org/resources.

### Phase 3: Piloting of the Case Studies

The five cases were piloted in an OPC *Special Education for Administrators Qualification Program* course. There were 109 participants in the course from 23 different school boards from across Ontario. The participants included principals and vice-principals from both elementary and secondary school settings. The course was offered online and links to each case were embedded in the materials of the course. Each case was presented in a different module of the course. Participants in the course responded to each case study as a course requirement. Response formats included asynchronous and synchronous discussion forums, written reflections, and video responses. Principals provided feedback on each case through an online questionnaire. The questionnaire solicited feedback on what participants founds to be effective or ineffective about the format and content of each of the cases.

The research team reviewed 258 responses to the cases and engaged in a thematic analysis of the data (Braun & Clarke, 2006). Similar to the Phase 1 data analysis, thematic analysis involved identifying frequently used words and phrases from the questionnaires (Braun & Clarke, 2006). From these data segments, categories of responses were developed. A validation activity was completed with the special education leader at the OPC who facilitated the course to review the anonymized feedback from participants and identify if this feedback aligned with this leader’s experiences and insights from coordinating the course.

## Findings

Analysis of the feedback from participants in the third phase of the study led to the following three key findings that we report on here: authenticity of the cases; importance of multiple perspectives; and, the effectiveness of the online format and branching scenarios.

### Authenticity

Principals indicated that the case studies were authentic and reflected real experiences in schools. According to the special education leader at the OPC, the cases were authentic because they reflected the complex and diverse situations that principals encounter with regards to supporting students with SEN. She said that the cases “are relevant to current experiences. When [principals] are reading them, they’re seeing themselves in those exact situations or they’ve heard about their colleagues being in similar situations.” Participants in the course described the cases as “authentic” and “relatable.” Specific feedback on cases included comments by two participants on Case Two [Ms. Lee’s Decision: A Case of Funding Changes for Students with Autism] in which they described it as “very real” and including “something that is happening in every school in Ontario.” Similarly, a participant confirmed the authenticity of Case Five [Ms. Morvan’s Decision: A Case of Student Oppositional Behaviour and Inclusive School Culture]: “I was able to relate as something similar is happening at my school site.”

Within this authenticity, however, respondents provided contrasting feedback on whether the cases provided enough details for participants to get a clear understanding of the diversity of issues in the case. The majority of respondents (82%) confirmed that the cases were simple, clear, concise, and provided a sufficient level of detail. For example, one respondent indicated that, “Enough details were shared to give you a clear understanding of the decisions that needed to be made.” A minority of respondents (18%), however, indicated that the cases should include further details about the diverse school and student contexts in the case. For example, although Case Five was considered “concise and direct” by one participant, this approach might not always reflect the reality of the processes with which principals must engage. One participant commented, “[T]he reality is though that the principal has to sift through information to get to the details.” Another principal stated, “It might have been helpful to have a little bit more background of the school [such as] demographic make-up.” This feedback reflects the complex role of principals who are often working in situations where they desire more information but have to make decisions with limited information.

### Multiple Perspectives

A majority of participants (79%) indicated that the case studies were effective because they presented multiple perspectives on the situations presented. The cases provided the perspectives of parents and other caregivers as well as teachers, students, and principals. An example of the type of feedback provided included one participant who commented about Case Five that, “The different perspectives presented were effective.”

The special education leader at the OPC indicated that providing multiple perspectives in cases authentically reflected the work principals engage in. Principals must consider multiple perspectives when they work through complex situations with students with SEN. The OPC leader said:

Every tricky situation that principals deal with needs to be disaggregated by role. How do I deal with the new teacher who is dealing with this situation? And the experienced teacher? And the parents? It reflects the complexity of how to lead.

Similarly, a course participant indicated that: “I like how [Case One] reflected the reality of the role of a school. The role of a principal, new teachers, [the] veteran (not really buying into inclusive education), [the] struggling student, and lack of parent involvement were all present in the case.” Another participant also expressed appreciation for the multiple perspectives in Case Five, exemplified by this comment: “I like how the content focused on reactions from staff, students, and families.” These different perspectives seemed to reflect the lived experiences of principals and provided nuanced and intersecting dimensions for consideration.

### Function, Form, and Choice

The vast majority (95%) of respondents indicated that the online, web-based interface of the cases was effective. All of the cases included an introductory narrative that presented the main “characters” in the cases and the prominent problems. Following these narratives, participants were presented with a webpage where they were able to select the principal’s response from a list of four options. After selecting a response, participants were presented with more information on the case and the ramifications of their selection. Participants were also presented with links to resources and videos where experienced principals provided a commentary on the case. Accessing these materials through a web-based interface allowed for easy access to multiple, branching scenarios and associated resources.

The majority of participants also found the task-by-task presentation an effective approach. One participant reflected on Case One, that “the case [is broken] into manageable chunks that allow individuals processing and reflection time before moving on to the next section.” Similarly, “Information was chunked so [it was] not overwhelming to read” was a comment on Case Two. One principal noted that, “dividing the case study into segments allowed for individuals to reflect on each section separately before having to look at the situation holistically.” The “ripple effect” of choosing a branching scenario and then being provided with further information mirrored what principals considered real-life situations where they do not have access to all of the information but gather this information – and adapt to it – as situations present themselves.

The “choose-your-own-adventure” style of branching scenarios appeared problematic for two participants. Related to Case One, one principal responded in detail:

The branching scenarios do not work. They are presented as though they would be separate options that could be taken when really they are all appropriate actions that should be taken. There is nothing wrong with this, but if you want to present it as options, there should be options that actually branch into different outcomes.

With a similar level of detailed response, another participant commented about Case Five that, “The branching actions really represented things that I would likely do simultaneously” and “I found that I became locked into a predetermined decision-making process that I would not have followed.” As these concerns emerged in Phase 3 study, a note was added to the website to specifically address these types of concerns related to the branching scenarios. The note clarified that the branches did not represent preferred options but potential pathways: “The case presents multiple options for your consideration, none of which may be explicitly correct or preferred.” This note helped participants better understand that there was no branching scenario that was the preferred option in the case.

## Discussion

In program development and design, Cosner (2019) suggests starting with a problem of practice and engaging those with experience in the problem in developing solutions for it. In a similar way, the team of university and educational partners commenced the study by considering how to effectively support the professional learning of school principals in relationship to leading inclusive schools for students with SEN. Similarly, in experiential learning theory, being able to engage with, reflect on, and make connections with concrete experiences is critical to changed practices (Kolb, 1984). Kolb argued that experiential learning provides a framework for the integration of analytical and socio-emotional elements of learning (Kolb & Fry, 1975). Online case studies provide a novel form of engagement in this learning process. Here we discuss the benefits of web-based case studies that this study has demonstrated, how web-based case studies can be effectively used for principals’ professional learning, and emerging questions for future research.

### The Benefits of Web-Based Case Studies

By engaging experienced school administrators in Phase 1 study, the research team was able to develop cases which were authentic and based on the real experiences of principals. In Phase 3 study, the input of more than 100 principals helped confirm that the focus of the cases, and the format of them via an online platform, was indeed authentic and highly accessible. The cases were viewed as rich learning tasks and participants identified the case studies as a valuable form of professional learning. This aspect aligns with recommendations for principals’ professional learning to focus on active learning which incorporates a significant amount of real-life experiences (Wang et al., 2018). Similarly, Tucker and Dexter (2011) identify a number of key benefits of online cases, which are reflected in the choose your own adventure form of professional learning from this study, including learner-determined pathways, real-time display of information, and scaffolding of decisions. Dexter et al. (2020) argue that,

The nonlinear structure of digital cases is more realistic in nature than a traditional narrative case, as a school and community present many sources of information that vary in relevance and ease of interpretation . . . this has the potential to overwhelm the learner, take them off track, or encourage muddling through, but such outcomes are also more authentic leadership experiences, which support transfer to real-life situations with similar dilemmas. (p. 184)

For those who lead principals’ professional learning, this approach to course development presents a novel and important way to create professional materials that are relevant and timely. Case studies can support critical thinking (Popil, 2011) and can help readers engage in a form of immersive learning (Sherman, 2008). Thus, case studies provide a form of experiential professional learning that can enhance learning by integrating the cognitive and the socio-emotional aspects of learning (Kolb, 1984). Web-based case studies appear to increase these types of benefits and also provide new benefits. Tucker and Dexter’s (2011) work demonstrates that online case studies can support procedural knowledge of new principals. As part of the development of this procedural knowledge, web-based case studies can foster principals’ understanding of diverse school contexts and the ramifications of decisions they make (Tucker & Dexter, 2011). Further, web-based case studies are highly accessible. The case studies for this study are openly available without cost to those who support principals’ professional learning. Since they are web-based, links to them are easily embedded in online learning materials. With increased attention to online and remote learning, web-based case studies provide a highly accessible form of learning.

Similarly, the accompanying flexibility that web-based case studies provide, allowing principals to engage in professional learning in a flexible-delivery fashion, can complement formal support that universities, professional organizations, and school boards can provide. Web-based case studies can be differentiated to offer different resources and pathways to account for the learners in courses. As Johnson et al. (2020) state:

When considering the type of support that school leaders need, another area of differentiated professional learning emerged: experienced leaders need altogether different professional learning opportunities than do less experienced leaders. . . the more experienced and higher skilled principals, the greater their need for differentiated professional learning opportunities. (p. 25)

Web-based case studies, for example, could provide more experienced principals with more complex and nuanced branching scenarios than pathways for less experienced principals.

The branching pathways with multiple options provided a sense of immersive and differentiated learning that provided some autonomy of control for the participant, another aspect of experiential learning theory (Hand et al., 2016). Having options for different pathways of choices that mirror professional decision-making is a key aspect of this immersive experience, thus resembling “choose your own adventure” options (Beck et al., 2017). This also supported the authenticity of the case studies as participants noted their resemblance to the real experiences they have in schools. Providing branching options can foster the development of ethical leadership practices which emerge “from a dynamic process where a principal can handle a similar ethical decision differently on different occasions” (DeMatthews & Serafini, 2019, p. 16). In their study of online cases, Tucker and Dexter (2011) describe that effective cases are

. . .designed to stimulate personal reflection through the reliance on real data or realistic events in all their complexity. There are no easy or right solutions to the tasks presented in the cases. This ambiguity is consistent with a primary goal of case methods, which is to understand that problems can be framed and solved in multiple ways. (p. 254)

The ambiguity of the cases, reflecting the complexity of the type of decisions that principals face on a daily basis, makes them an effective tool for experiential learning.

A further benefit of web-based case studies for principal professional learning is that changes can be made to the case studies based on the feedback presented by participants. For example, in this study, the branching pathways were initially identified as confusing by some participants because they were looking for preferred pathways. Clarifying communication about the format as feedback was received demonstrated nimble, “real-time” adaptation. Contrasted with case studies that are written and in books, this form of case study provides an innovative form of professional learning (Matcham et al., 2016).

It should also be noted that the branching scenarios of web-based case studies requires those who complete them to engage in critical self-reflection due to the diverse range of options that they provide (Beck et al., 2017). This is particularly important for experiential learning related to inclusion as critical reflection is a “mental habit developed during preparation that supported leading for inclusion” (DeMatthews et al., 2020, p. 19). Web-based case studies are a form of problem-based learning that incorporate “high-impact leadership preparation strategies” (DeMatthews et al., 2020, p. 21) that reflect Kolb’s (1984) focus on experiential learning.

### Five Lessons for Developing Web-Based Case Studies

As others consider web-based case studies in education, it is important to note that these types of novel case studies have been effectively used in other professions such as medicine (Beck et al., 2017; Burke, 2017; Matcham et al., 2016). Here we provide five lessons learned that can help others in their development of web-based case studies: authenticity, accessibility and adaptability, complexity, multiple perspectives, and resource rich.

#### Authenticity

First, web-based case studies need to be developed which authentically represent problems of practice (Cosner, 2019; Wang et al., 2018). The participants in this study noted that one of the defining aspects of the case studies was that they were based on actual events and authentically reflected their lived experiences. Developing the cases from the insights of experienced principals ensured that the cases represented the types of situations that principals experience in their work with students with SEN.

#### Accessibility and adaptability

Second, web-based case studies provide a high degree of accessibility and adaptability (Burke, 2017). In other words, they can be adjusted and differentiated even after being published. This ensures that cases remain timely and reflect current realities that school principals encounter. In this study, changes to cases—such as providing clarifying instructions—were easily able to be made after the initial launch of the online cases. Similarly, new materials could be easily added to reflect changing policies, procedures, and resources.

#### Complexity

Third, cases need to be sufficiently complex so as to resemble real-life scenarios and options, especially if the participants completing the case studies are experienced professionals such as principals (Wang et al., 2018). The complexity of case studies is heightened when concrete emotional experiences are incorporated, thus leading to the “labeling or relabeling of immediate existential experience” (Kolb & Fry, 1975, p. 34). This process enables participants to see themselves within the case while also allowing them to remain detached in order to engage in analytical processes. Consideration for the needs of new principals—who may not have the same degree of professional knowledge (Slater et al., 2018)—also needs to be given (Matthews & Crow, 2010; Tucker & Dexter, 2011). Further, cases need to represent the diversity of student identities and school contexts (DeMatthews et al., 2021). In other words, the cases needs to be sufficiently complex, but not too convoluted, for a range of participants.

#### Multiple perspectives

Fourth, web-based case studies need to include enough multiple perspectives and complicating elements that allows principals to sift through information and prioritize and attend to relevant details for decision-making (Beck et al., 2017). Providing diverse perspectives of students, family members, teachers, support staff, system leaders, and principals can enrich the case study. This also ensures that the “voices” of these constituent groups are considered in the identification of problems and determination of next steps.

#### Resource rich

Fifth, we would encourage the use of multiple modes of resources to support the case studies including videos and web-links. These supporting resources were highly valued by the participants in this study. Using a web-based platform allows ease of access to these resources and also facilitates different modalities of learning including auditory and visual. Tucker and Dexter’s (2011) Educational Theory Into Practice Software (ETIPS) even provides opportunities for participants to design their own case studies, thus providing a rich, authentic, and differentiated experience.

### Limitations and Questions for Future Research

There are a number of limitations to this study. First, the study does not explore if the web-based case studies influence principals’ behavior in supporting inclusive schools. We recommend future research to incorporate standardized scales before and after completing cases to assess principals’ learning and impact on practice (Tucker & Dexter, 2011). In addition to the connections we have raised to Kolb and experiential learning theory, further considerations for experiential learning and adult learning theory would inform this future research. For example, we have not considered participants’ learning preferences, such as those who examine experiences from divergent perspectives or those who rely more on intuition than analysis. Second, the study uses multiple pathways for principals to pursue in each case without providing preferred or “correct” options. Further research could provide “choose your own adventure” pathways which incorporate preferred and less preferred options, thus providing a more challenging opportunity for principals to use procedural knowledge to identify effective practices. Third, the study is limited in the feedback that was provided by the participants in the third phase. Participants in this phase used a simple questionnaire at the conclusion of each case to provide feedback. Including interview data in this phase would have provided enhanced participant input.

Although the focus of this study was not on how the web-based case studies influenced principals’ practices in supporting students with SEN, it does lead us to consider further questions for research in this area. For example, in what ways could web-based case studies contribute to what we know about inclusion and professional learning? Research on the use of immersive technologies in this field needs to support learning for both principals and teachers as a “collective property of the school, to which many contribute and from which many will benefit.” (Dexter & Richardson, 2020, p. 33). Given that there is generally a lack of scholarly literature at the intersection of inclusion and school leadership, more research in this area would be welcomed (Cobb, 2015; DeMatthews et al., 2021; Sider, 2020; Sider et al., 2017). Research that explores how immersive case studies can support professional learning related to the intersectionalities of students with SEN would also be welcomed since “much of the leadership literature focuses on how well-prepared principals create inclusive schools without consideration of race and other forms of student identity” (DeMatthews et al., 2021, p. 10). Further, in what ways can web-based case studies contribute to what we know about the changing way principals learn? As online learning prospects increase, the web-based case studies developed in this study present a new opportunity for research in principals’ professional development.

## Conclusion

This study provided an opportunity to explore the process of developing authentic learning resources for principals’ professional learning. The research partners engaged experienced principals to help identify key problems of practice regarding inclusion of students with SEN. The input from experienced principals led to the development of five web-based case studies on inclusive school leadership that are openly accessible. These case studies were used with more than 100 participants in a professional learning course on leadership and special education. The OPC has identified other courses and workshops on school leadership and special education for which the case studies will serve as a supplementary resource. This demonstrates a multiplication effect in which the case study resources can be used for multiple purposes and audiences.

The process of developing web-based case studies that were used in this study can serve as a model for effective practice as others consider similar authentic learning tools to support principals’ professional learning. Developing the case studies based on the input of experienced principals ensured that the narratives were realistic and perceived as such by course participants. Branching scenarios offered multiple perspectives and options to the participants, thus representing the day-to-day experiences, decisions, and choices of principals. The online format of the case studies provided a form of learning that was seen as functional and flexible by the participants and offered choice in the form of “choose your own adventure” which may not have been available in a traditional, text-based case study. These web-based case studies may be particularly effective for supporting professional learning related to inclusion because of the need for critical reflection and a level of experiential learning. These benefits of web-based case studies are significant and provide an opportunity to consider further research in this and other new forms of professional learning for principals.

As others develop web-based case studies as a form of professional learning, we would encourage them to ensure that the case studies are authentic, accessible and adaptable, sufficiently complex, offer multiple perspectives, and are linked to other resources. The process of developing web-based case studies that is outlined here can serve as an effective model to meet the professional learning needs of principals while also addressing the scholarly need for further research on how these forms of learning can be effectively developed in the future.

## Appendix: Screen shots of the cases

Case Options at www.leadtoinclude.org

## Notes

1. We refer to those in formal positions of administrative school leadership as “principals” recognizing that this includes vice-principals.
